# Antiseptic efficacy of an innovative perioperative surgical skin preparation: A confirmatory FDA phase 3 analysis

**DOI:** 10.1017/ice.2020.27

**Published:** 2020-06

**Authors:** Charles E. Edmiston, Philip Lavin, Maureen Spencer, Gwen Borlaug, Gary R. Seabrook, David Leaper

**Affiliations:** 1 Department of Surgery, Medical College of Wisconsin, Milwaukee, Wisconsin, United States; 2 Boston Biostatistics Research Foundation, Framingham Massachusetts, United States; 3 Infection Preventionists, Boston, Massachusetts; 4 Borlaug Infection Prevention Services, Inc., Phoenix, Arizona, United States; 5 Department of Clinical Sciences, University of Huddersfield, Huddersfield, United Kingdom

## Abstract

**Background:**

An innovative approach to perioperative antiseptic skin preparation is warranted because of potential adverse skin irritation, rare risk of serious allergic reaction, and perceived diminished clinical efficacy of current perioperative antiseptic agents. The results of a confirmatory US Food and Drug Administration (FDA) phase 3 efficacy analysis of a recently approved innovative perioperative surgical skin antiseptic agent are discussed.

**Methods:**

The microbial skin flora on abdominal and groin sites in healthy volunteers were microbiologically sampled following randomization to either ZuraGard, a 2% chlorhexidine/70% isopropyl alcohol preparation (Chloraprep), or a control vehicle (alcohol-free ZuraGard). Mean log_10_ reduction of colony-forming units (CFU) was assessed at 30 seconds, 10 minutes, and 6 hours.

**Results:**

For combined groin sites (1,721 paired observations) at all time points, the mean log_10_ CFU reductions were significantly greater in the ZuraGard group than in the Chloraprep group (*P* < .02). Mean log_10_ CFU reductions across combined abdominal and groin sites at all time points (3,277 paired observations) were significantly greater in the ZuraGard group than in the Chloraprep group (*P* < .02).

**Conclusions:**

A confirmatory FDA phase 3 efficacy analysis of skin antisepsis in human volunteers documented that ZuraGard was efficacious in significantly reducing the microbial burden on abdominal and groin test sites, exceeding that of Chloraprep. No significant adverse reactions were observed following the application of ZuraGard.

**Trial registration:**

ClinicalTrials.gov identifiers: NCT02831998 and NCT02831816.

Approximately 45 million surgical procedures are performed each year in the United States, and a conservative estimate of 500,000 of these procedures result in a surgical site infection (SSI).^1,2^ A multistate point-prevalence survey published in 2014 found that SSIs represented one of the most common healthcare-associated infections (21.8%).^3^ Surgical site infections lead to increased length of hospital stays, higher rates of unplanned reoperation, higher rates of hospital readmission, and a 2- to 4-fold higher risk of death.^2–9^ Annually, SSIs may result in 10,000 avoidable deaths and $9 billion in excess healthcare costs in the United States.^10,11^

The patients’ endogenous microflora, primarily skin-colonizing organisms, are responsible for a significant proportion of SSIs. Perioperative skin antisepsis decreases the number of bacteria colonizing the skin, thereby reducing the risk of contamination of the incisional wound, and it is viewed as the sentinel interventional risk-reduction strategy by the World Health Organization, the Centers for Disease Control and Prevention, the National Institute Center for Health and Care Excellence, and The American College of Surgeons and Surgical Infection Society.^12–16^

Two major classes of perioperative antiseptic skin preparations are used in the United States: chlorhexidine gluconate (CHG)-based or iodine (iodophor)-containing antiseptic agents. Current guidelines recommend the use of a perioperative antiseptic skin preparation that contains alcohol, which has an immediate impact on reducing the microbial burden. When combined with an additional agent, such as CHG or an iodophor, residual antiseptic activity results for the duration of the surgical procedure.^17–20^

Products containing 70% isopropyl alcohol with 2% chlorhexidine gluconate (CHG) are widely used as a perioperative topical skin preparation in the United States. CHG is active against most common gram-positive and gram-negative surgical wound pathogens. However, its widespread use has been viewed as a potential risk for the emergence of resistance pathogens, which may impact its future utility.^21–24^ In addition, skin irritation and rare allergic reactions have been reported with antiseptic products containing CHG.^25^ Therefore, continued development of new, safe, and clinically effective antiseptic formulations are warranted.

ZuraGard is a 70% isopropyl alcohol–based antiseptic formulation with functional excipients, citrate (ie, citric acid and sodium citrate), and alkyl parahydroxybenzoates (with methylene blue as a colorant). The initial antimicrobial efficacy and safety profile for ZuraGard has been assessed in two phase 2 studies involving 96 evaluable healthy participants.^26^ The agent expressed immediate and persistent antimicrobial activity for up to 24 hours with no skin irritation or other adverse events. The current manuscript reports the findings from a large phase 3 efficacy study conducted according to the 2018 US Food and Drug Administration (FDA) Final Rule, Safety and Effectiveness for Healthcare Antiseptics criteria to assess the efficacy of a novel skin antiseptic on both abdominal and groin skin sites in >800 healthy human participants.^27^

## Materials and methods

### Study design

Two phase 3 randomized studies were combined for analysis. In each study, participants were randomized to 3 groups: (1) ZuraGard versus, (2) Chloraprep versus a control, or (3) ZuraGard versus Chloraprep. The present study presents results for the paired ZuraGard versus Chloraprep comparison, which is based solely on the third group. Data for 2 locations (groin and abdomen) were collected for all study participants at 3 separate times: 30 seconds, 10 minutes, and 6 hours.

### Study sites

The antiseptic efficacy study involving an innovative preoperative antiseptic agent ZuraGard (Zurex Pharma, Middleton, WI) was conducted at 2 separate test laboratories: MicroBiotest, designated as Z73 (Sterling, VA) and BioScience Laboratories, designated as Z74 (Bozeman, MT). Identical study protocols were reviewed and approved by 2 separate independent institutional review boards (MicroBioTest Laboratories and BioScience Gallatin Institutional Review Boards) prior to participant recruitment. The study was performed according to the 2018 FDA Final Rule, Safety and Effectiveness of Healthcare Antiseptics.^27^ The study protocol was approved by the FDA and registered on ClinicalTrials.gov. The ClinicalTrials.gov identifier for the MicroBio Test study is NCT02831998. The identifier for the BioScience Laboratory study is NCT02831816.

### Inclusion/Exclusion criteria

Participants eligible for enrollment in this study met the following criteria:

- Healthy male or female volunteer, 18 years of age or older (participants aged <18 years must have written custodial consent)
- Good general health
- Skin within 15.25 cm (6 inches) of the test sites that is free of tattoos, dermatoses, abrasions, cuts, lesions or other skin disorders
- Cooperative and willing to follow the participant instructions
- Cooperative and willing to sign consent form and HIPAA authorization form
- Screening day baseline counts of at least 1.0 × 10^3^ CFU/cm^2^ per abdominal site (left and right) and at least 1.0 × 10^5^ CFU/cm^2^ per groin site (left and right). For replacement participants, screening day baseline counts of at least 1.0 × 10^3^ CFU/cm^2^ per abdominal site (left and right) and/or at least 1.0 × 10^5^ CFU/cm^2^ per groin site (left and right).

Participants with any of the following conditions were excluded from this study:

- Topical or systemic antimicrobial exposure within 14 days prior to screening day. Restrictions include, but are not limited to antimicrobial soaps, antiperspirants/deodorants, shampoos, lotions, perfumes, after shaves, colognes, and topical or systemic antibiotics
- Swimming in chemically treated pools or bathing in hot tubs, spas and whirlpools within 14 days prior to screening day
- Use of tanning beds, hot waxes, or depilatories, including shaving (in the applicable test areas) within 14 days prior to screening day
- Contact with solvents, acids, bases, fabric softener-treated clothing or other household chemicals in the applicable test areas within 14 days of the screening day
- History of sensitivity to natural rubber latex, adhesive skin products (eg, Band-Aids, medical tapes), isopropyl alcohol, citric acid, methylene blue, methylparaben, propylparaben, or CHG products
- History of skin allergies
- History of skin cancer within 15.25 cm (6 inches) of the applicable test areas
- Pregnant, attempting pregnancy or nursing (For all females of child-bearing potential (aged <60 years), a pregnancy test was performed before treatment on treatment day.)
- Showered or bathed within 72 hours of the screening day or treatment day (Sponge baths may be taken; however, the lower abdomen and upper thigh region must be avoided).
- Received an irritation score of 1 for any individual skin condition prior to the screening day baseline or treatment day baseline sample collection
- Participation in another clinical trial in the 30 days prior to test day of this study (treatment with test materials in this study) or currently enrolled in another clinical trial or previously participated in this study

In total, 2,159 participants were screened for study inclusion; 1,080 (~50%) passed screening baseline and were treated; 966 (44.7%) were included who passed treatment day minimal microbial skin burden baselines. The most common reason for participant exclusion was failure to present with the minimal treatment day baseline microbial burden required to validate antiseptic efficacy according to FDA guidelines.

### Study logistics

The goal of each phase 3 efficacy study was to assess the immediate and persistent activity of ZuraGard against endogenous bacterial flora on the skin of adult participants, comparing this antimicrobial activity to the comparator agent Chloraprep, 70% alcohol/2% chlorhexidine gluconate (Becton Dickinson, Franklin Lakes NJ), and a negative control, designated as the alcohol-free ZuraGard negative control vehicle.

On the day of treatment, following informed consent, participants were randomized to 1 of the 3 possible treatment pairs to be tested: the alcohol-free ZuraGard vehicle versus ZuraGard, the alcohol-free ZuraGard vehicle versus Chloraprep, and ZuraGard versus Chloraprep. Both the groin and abdomen locations were tested (left vs right). Participants underwent microbiological sampling to measure baseline cutaneous microbial counts at abdominal and groin sites prior to application of the study test solutions. Participants were also randomized to receive 2 of the 3 possible treatments (1 per side). In each study volunteer, groin and abdominal sites on each side (left compared with right) were randomly assigned to 1 of the allotted 2 treatment agents. Test solutions were applied in a standardized manner by trained research personnel familiar with perioperative skin-prepping technique. The comparative agents were applied by “scrubbing” over a 3.8 × 12.7 cm (1.5 × 5 inches) area at groin sites and over a 12.7 × 12.7 cm (5 × 5 inches) area at abdominal sites. Each anatomic test site was allowed to air dry for 3 minutes, and after treatment the weight of the antiseptic applicators was recorded to ensure a uniform application volume. Following the 10-minute microbiological sampling, abdominal and groin sites were covered with a semiocclusive bandage, and volunteers were instructed not to touch or remove the dressing prior to the 6-hour skin sampling collection. Cutaneous microbiological samples were collected at 30 seconds, 10 minutes, and 6 hours according to FDA criteria.^27^

At both study sites, the primary measure was based on mean log_10_ reductions in CFUs/cm^2^ from baseline for ZuraGard, Chloraprep, and the alcohol-free ZuraGard negative control vehicle. On abdominal sites, the primary measure achieved a ≥2 log_10_ CFU/cm^2^ reduction from baseline at 30 seconds and 10 minutes following application of the test solution and maintained a log reduction below baseline for 6 hours. On the groin sites, the primary measure achieved a ≥3 log_10_ CFUs/cm^2^ reduction from baseline at 30 seconds and 10 minutes after application of the test solution and maintained a log-reduction level below baseline for 6 hours. To utilize data across all testing sites, the protocol analysis focused on the mean log_10_ reduction from baseline for the groin and abdominal skin surfaces, separately and in a combined analysis. The final number of participants tested with each treatment is summarized in Table 1 by location and time per study; these counts excluded treatment-day baseline failures. The final number of ZuraGard–Chloraprep pairs is summarized in Table 2. In total, 832 participants (Z3: 357, Z4: 475) contributed ZuraGard data and 844 (Z3:355, Z4:489) contributed to Chloraprep data. By combining paired treatment data for both studies across all 3 observation times, a total of 1,721 ZuraGard–Chloraprep pairs were collected from groin sites and 1,556 ZuraGard–Chloraprep pairs were collected from abdominal sites.

**Table 1.** Participant Treatment Counts Over Time Per Location Per Study

Location	Time	No. of Individual Participants per Anatomic Location					
		Study Sites^a^					
		Z3			Z4		
		ZuraGard	Chloraprep	Alcohol-Free ZuraGard Vehicle^a^	ZuraGard	Chloraprep	Alcohol-Free ZuraGard Vehicle^b^
Groin	30 s	330	326	68	343	352	74
	10 min	330	326	68	342	352	74
	6 h	330	326	68	343	351	74
Abdomen	30 s	342	340	69	324	320	68
	10 min	342	340	69	324	320	68
	6 h	342	340	69	323	319	68
Either	30 s	357	355	71	475	489	110
	10 min	357	355	71	474	489	110
	6 h	357	355	71	474	488	110

a Z3 = MicroBiotest Inc; Z4 = BioScience Laboratories.

b Alcohol-free ZuraGard negative control vehicle data were not used in the comparative data analysis.

**Table 2.** Participant Pairs (ZuraGard and Chloraprep) Over Time Per Location Per Study

		Pair Counts		
		Study Sites^a^		
Location	Time	Z3	Z4	Z3+Z4
Groin	30 s	293	281	574
	10 min	293	281	574
	6 h	293	280	573
	Combined	879	842	1,721
Abdomen	30 s	305	214	519
	10 min	305	214	519
	6 h	305	213	518
	Combined	915	641	1,556
Either	30 s	319	378	697
	10 min	319	378	697
	6 h	319	377	696
	Combined	957	1,133	2,090
Combined	30 s	598	495	1,093
	10 min	598	495	1,093
	6 h	598	493	1,091
	Combined	1,794	1,487	3,277

Note. Mixed model based on participant counts using all locations over all times.

a Z3 = MicroBiotest Inc; Z4 = BioScience Laboratories.

### Bacterial skin surface sampling

Microbial specimens were collected from each skin site using a sterile cylinder containing 3.0 mL sterile stripping solution with product neutralizers (eg, peptone, egg lecithin, histamine hydrochloride, sodium chloride, potassium dihydrogen phosphate, and disodium hydrogen phosphate dihydrate). Once in contact with the skin, the area surrounding the cylinder was massaged to enhance collection of cutaneous flora. All samples were transferred to a sterile counting tube. A second aliquot sample was collected in the same manner immediately following the first sample. Both were combined and serially diluted in Butterfield’s phosphate buffer containing product neutralizers. Plated cultures were prepared from each of these dilutions on tryptic soy agar with product neutralizers (TSA+) and incubated at 30°C for 72 hours to allow for sufficient bacterial growth. The origin of all samples were blinded to individuals determining colony counts under visual magnification. Cutaneous bacterial counts at baseline and each postapplication sampling period (30 seconds, 10 minutes, and 6 hours) were recorded for each skin-surface test site (log_10_ CFU/cm^2^).

### Statistical analysis

A modified intent-to-treat (mITT) approach was used for statistical analysis, restricted to participants with baseline cutaneous microbial counts >3.1 log_10_ CFU/cm^2^ at abdominal sites and >5.6 log_10_ CFU/cm^2^ at groin sample sites on the day of treatment. Differences in counts between baseline and each programmed postapplication period were calculated as log_10_ CFU/cm^2^ data. Descriptive statistics (ie, mean, median, standard deviation (SD), and minimum/maximum recovery) were computed for each sampling site and for the postapplication test periods. The results for study sites designated Z3 and Z4 were combined for analysis.

The study analysis was based on the mean paired difference between ZuraGard and Chloraprep at all 3 time intervals and both anatomical sample site locations among participants receiving both active treatments. The log_10_ CFU/cm^2^ differences from baseline paired treatments were combined for the abdominal and groin sites. A negative sign favored ZuraGard over Chloraprep. Any paired data from either site at any time were included in the analysis. Participants randomized to receive the negative control (ie, the alcohol-free ZuraGard vehicle) could only receive 1 of the 2 active treatments: ZuraGard or Chloraprep. Therefore, data for the participants treated with the alcohol-free ZuraGard vehicle were excluded from the paired ZuraGard and Chloraprep efficacy analysis.

The primary data analyses were based on a longitudinal model (SAS proc mixed procedure) with terms for location (ie, groin or abdomen), time (30 seconds, 10 minutes, or 6 hours), and study. At each time, there were 4 possible outcomes for each participant (2 locations × 2 treatments per location). The model assumed an unstructured covariance matrix to estimate effects. The combination of the 2 studies was preplanned. The following testing sequence was preset according to a hierarchical testing plan: (1) to first rule out study differences, (2) to then compare the combined locations across both studies, (3) to then compare just the groin location across both studies, and (4) to finally compare the abdomen location across both studies. To preserve the type I error for multiple comparisons, a 2-sided *P* < .025 was required to achieve statistical significance for each test given the separate groin and abdomen testing. All results were reported as a net change (log scale) and 95% confidence intervals (95% CI) calculated using the least square means from the respective models. SAS version 9.2 statistical software (SAS Institute, Cary, NC) was used for all analyses.

### Participant safety

Adverse reactions were monitored by the study personnel at each test site over a 6-hour period. All local and systemic adverse events observed or reported to the investigators (ie, mild skin irritation, erythema, or skin allergic reactions) were evaluated and followed to resolution along with intensity, duration, and causal relationship to the tested agent.

## Results

The final number of participants tested with each treatment is summarized in Table 1 by location and time per study; these counts excluded baseline failures. The final number of ZuraGard–Chloraprep pairs is summarized in Table 2. In total, 832 participants (Z3, 357; Z4, 475) contributed ZuraGard data and 844 (Z3, 355; Z4, 489) contributed to Chloraprep data. By combining paired treatment data for both studies across all 3 observation times, 1,721 ZuraGard–Chloraprep pairs were collected from groin sites and 1,556 ZuraGard–Chloraprep pairs were collected from abdominal sites.

Log-reduction patterns for ZuraGard and ChloroPrep and the alcohol-free ZuraGard negative control vehicle were similar across both studies. Table 3A–C lists the mean log_10_ CFU/m^2^ reductions for the paired comparisons of the alcohol-free ZuraGard vehicle versus ZuraGard and the alcohol-free ZuraGard vehicle versus Chloraprep. Both active treatment groups ZuraGard and Chloraprep outperformed the alcohol-free ZuraGard vehicle, as expected. Table 4A–C documents the mean log_10_ CFU/m^2^ reductions for the direct paired comparisons of ZuraGard versus Chloraprep. The ZuraGard log reductions across all anatomical sites and time points were consistently higher than those for Chloraprep in both studies.

**Table 3A.** Mean Log_10_ CFU/cm^2^ Reduction From Baseline With 95% Confidence Intervals at 30 Seconds Post Application

Study^a^	Abdomen			Groin		
	Vehicle Mean (95% CI)	ZuraGard Mean (95% CI)	ChloraPrep Mean(95% CI)	Vehicle Mean (95% CI)	ZuraGard Mean (95% CI)	ChloraPrep Mean (95% CI)
ZX-73	1.07 (0.93–1.21)	3.04 (2.73–3.35)	2.76 (2.48–3.03)	1.59 (1.42–1.75)	3.97 (3.63–4.32)	3.88 (3.44–4.33)
ZX-74	0.71 (0.56–0.86)	2.90 (2.54–3.25)	2.56 (2.20–2.92)	1.23 (1.07–1.39)	3.97 (3.49–4.45)	3.87 (3.42–4.33)

Note. CFU, colony-forming units; CI, confidence interval.

a Studies ZX-73 and ZX-74: only vehicle pairs.

**Table 3B.** Mean Log_10_ CFU/cm^2^ Reduction From Baseline With 95% Confidence Intervals at 10 Minutes Post Application

Study^a^	Abdomen			Groin		
	Vehicle Mean (95% CI)	ZuraGard Mean (95% CI)	ChloraPrep Mean (95% CI)	Vehicle Mean (95% CI)	ZuraGard Mean (95% CI)	ChloraPrep Mean (95% CI)
ZX-73	1.46 (1.31–1.61)	3.41 (3.19–3.64)	3.27 (3.12–3.42)	2.21 (2.02–2.40)	4.84 (4.57–5.11)	4.62 (4.30–4.94)
ZX-74	1.00 (0.78–1.21)	2.74 (2.37–3.11)	2.84 (2.52–3.16)	1.60 (1.44–1.76)	4.19 (3.76–4.62)	4.24 (3.80–4.68)

Note. CFU, colony-forming units; CI, confidence interval.

a Studies ZX-73 and ZX-74: only vehicle pairs.

**Table 3C.** Mean Log_10_ CFU/cm^2^ Reduction From Baseline With 95% Confidence Intervals at 6 Hours Post Application

Study^a^	Abdomen			Groin		
	Vehicle Mean (95% CI)	ZuraGard Mean(95% CI)	ChloraPrep Mean(95% CI)	Vehicle Mean (95% CI)	ZuraGard Mean (95% CI)	ChloraPrep Mean (95% CI)
ZX-73	1.18 (1.00–1.37)	2.61 (2.22–3.01)	2.47 (2.09–2.85)	2.02 (1.79–2.24)	2.72 (2.35–3.09)	3.09 (2.75–3.44)
ZX-74	1.37 (1.10–1.64)	2.94 (2.61–3.28)	2.80 (2.43–3.16)	2.10 (1.91–2.28)	4.17 (3.69–4.64)	3.94 (3.62–4.27)

Note. CFU, colony-forming units; CI, confidence interval.

a Studies ZX-73 and ZX-74: only vehicle pairs.

**Table 4A.** Mean Log_10_ CFU/cm^2^ Reduction From Baseline With 95% Confidence Intervals at 30 Seconds Post Application

Study^a^	Abdomen		Groin	
	ZuraGard Mean (95% CI)	ChloraPrep Mean (95% CI)	ZuraGard Mean (95% CI)	ChloraPrep Mean (95% CI)
ZX-73	3.00 (2.89–3.10)	2.91 (2.80–3.02)	3.91 (3.77–4.06)	3.82 (3.67–3.97)
ZX-74	2.74 (2.61–2.88)	2.76 (2.62–2.90)	3.80 (3.65–3.95)	3.78 (3.62–3.93)

Note. CFU, colony-forming units; CI, confidence interval.

a Studies ZX-73 and ZX-74: only ZuraGard and Chloraprep pairs.

**Table 4B.** Mean Log_10_ CFU/cm^2^ Reduction From Baseline With 95% Confidence Intervals at 10 Minutes Post Application

Study^a^	Abdomen		Groin	
	ZuraGard, Mean (95% CI)	ChloraPrep, Mean (95% CI)	ZuraGard, Mean (95% CI)	ChloraPrep, Mean (95% CI)
ZX-73	3.36 (3.29–3.43)	3.36 (3.29–3.43)	4.83 (4.72–4.95)	4.80 (4.68–4.92)
ZX-74	3.02 (2.89–3.15)	2.93 (2.80–3.06)	4.02 (3.87–4.17)	3.98 (3.82–4.14)

Note. CFU, colony-forming units; CI, confidence interval.

a Studies ZX-73 and ZX-74: only ZuraGard and Chloraprep pairs.

**Table 4C.** Mean Log_10_ CFU/cm^2^ Reduction From Baseline With 95% Confidence Intervals at 6 Hours Post Application

Study^a^	Abdomen		Groin	
	ZuraGard Mean(95% CI)	ChloraPrep Mean (95% CI)	ZuraGard Mean (95% CI)	ChloraPrep Mean (95% CI)
ZX-73	2.44 (2.32–2.55)	2.43 (2.31–2.54)	3.04 (2.92–3.15)	2.98 (2.87–3.09)
ZX-74	3.04 (2.92–3.16)	2.98 (2.87–3.09)	3.94 (3.80–4.07)	3.79 (3.64–3.95)

Note. CI, confidence interval.

a Studies ZX-73 and ZX-74: only ZuraGard and Chloraprep pairs.

Table 5 presents the mean paired differences for each location and time for each study individually and combined; 11 of the 12 possible individual comparisons demonstrated an advantage for ZuraGard over Chloraprep. Table 6 presents the preplanned tests from the longitudinal model for the abdomen and groin data combined across studies for the 3 time points as well as for the individual and combined locations. In both tables, a negative sign favors ZuraGard. For the combined groin sites and time points across both studies, the mean reduction in the ZuraGard group was significantly greater than in the Chloraprep group (2-sided *P* < .02). For all combined sites and time points across studies, the mean reduction was also significantly greater in the ZuraGard group (2-sided *P* < .02).

**Table 5.** Observed Mean Log-Reduction Differences (ZuraGard–Chloraprep) Over Time Per Anatomic Location Per Investigational Study Site

Location	Time	Mean Paired Differences^a^		
		Study Sites^b^		Combined
		Z3	Z4	Z3+Z4
Groin	30 s	−0.089	−0.044	−0.067
	10 min	−0.038	−0.095	−0.066
	6 h	−0.055	−0.165	−0.108
	Combined	−0.06	−0.101	−0.081
Abdomen	30 s	−0.09	0.033	−0.039
	10 min	−0.005	−0.079	−0.035
	6 h	−0.009	−0.003	−0.006
	Combined	−0.034	−0.038	−0.027
Combined	30 s	−0.09	−0.011	−0.054
	10 min	−0.02	−0.088	−0.051
	6 h	−0.031	−0.095	−0.06
	Combined	−0.047	−0.065	−0.055

a Negative sign favors ZuraGard.

b Z3 = MicroBiotest; Z4 = BioScience Laboratories.

**Table 6.** Model-Based Mean Differences in Change From Baseline Log_10_ CFU

Paired Difference (ZuraGard–Chloraprep) in Change from Baseline^a^				
Study Site^b^	No. of Differences	Location	Least Squares Mean (95% CI)	*P* Value
Z3+Z4	1,556	Abdomen	−0.027 (−0.083 to 0.029)	.3494
	1,721	Groin	−0.081 (−0.145 to −0.018)	.0123
	3,277	Combined	−0.053 (−0.096 to −0.014)	.0149

Note. CFU, colony-forming units; CI, confidence interval.

a Negative sign favors ZuraGard.

b Z3 = MicroBiotest; Z4 = BioScience Laboratories.

### Safety

No significant adverse events were noted in the 3 treatment arms (Chloraprep, the alcohol-free ZuraGard vehicle, and ZuraGard) at each of the 3 time points (30 seconds, 10 minutes, and 6 hours).

## Discussion

ZuraGard contains isopropyl alcohol as an active ingredient as well as the functional excipients citrate (citric acid and sodium citrate) and alkyl parahydroxybenzoates (with methylene blue as a colorant). In the previously published phase 2 studies, ZuraGard was compared with Chloraprep (active control) and the alcohol-free ZuraGard vehicle (negative control) to assess the immediate and persistent activity of ZuraGard against endogenous bacterial skin flora. ZuraGard, Chloraprep, and the alcohol-free ZuraGard vehicle were applied to the abdomen (which contains few sebaceous glands) and groin (which is rich in sebaceous glands), baseline and postapplication skin microbiological samples were obtained and cultured. At 10 minutes after ZuraGard application, cutaneous microbial counts dropped by ~3 log_10_ CFU/cm^2^ on both groin and abdominal sites. This reduced microbial burden was maintained at the groin site for 24 hours after application. A 2 log_10_ CFU/cm^2^ reduction was observed at 24 hours on abdominal test sites following application of the ZuraGard antiseptic agent.^26^

Reducing the microbial burden on skin prior to a surgical procedure is an effective strategy for reducing the risk of SSI.^17,18,20^ Existing guidelines recommend alcohol-containing products to prepare the skin prior to any surgical procedure because alcohol-containing antiseptic agents act more rapidly than an aqueous agent.^12–16^ In the present FDA phase 3 efficacy analysis, the results demonstrate the effectiveness of a novel perioperative skin antiseptic, ZuraGard, in reducing bacterial counts on the surface of healthy volunteers. ZuraGard reduced microbial counts at both groin (≥3 log_10_ CFU/cm^2^ reduction) and abdominal (≥2 log_10_ CFU/cm^2^ reduction) sites, caused minimal skin irritation or other adverse events, and ZuraGard performed better than Chloraprep, which is currently the standard-of-care perioperative antiseptic skin preparation in the United States. Unlike previous studies with Chloraprep, the FDA analysis required ZuraGard to achieve targeted microbial log reductions at a more challenging 30 seconds after application. In addition, targeted log-reductions were achieved at 10 minutes after application with microbial reduction were sustained for up to 6 hours (per FDA criteria).

In view of the potential risk of bacterial wound contamination during a surgical procedure and given the short duration of alcohol-based antiseptic activity, current guidelines recommend that agents used for surgical site preparation also contain an additional component to promote prolonged residual antimicrobial activity. Although CHG possess residual activity that is more pronounced than iodophors, with the notable exception of the recent National Institute for Health and Care Excellence (NICE) guidelines, there is insufficient evidence to preferentially support one type of alcohol-containing preparation over another or to suggest that the addition of another antimicrobial could contribute to efficacy.^14,19,20,28,29^

ZuraGard is a 70% isopropyl alcohol-based antiseptic formulated with functional excipients citrate (citric acid and sodium citrate) and alkyl parahydroxybenzoates that support the activity of alcohol, helping to maintain the persistent antimicrobial activity of the antiseptic agent. In the formulation presented in this study, methylene blue functions as a colorant, but the formulation is also available in an orange tint and in a colorless formulation. There are pragmatic reasons for the continued development and evaluation of alternatives to conventional preoperative skin antiseptic agents. For example, adverse events reported in the FDA Adverse Event Reporting System document IgE-mediated anaphylactic/anaphylactoid reactions triggered by CHG exposure and, although they are very rare, they are increasingly reported in association with surgical procedures.^30^ Hypersensitivity reactions mediated by other mechanisms have also been recognized.^31^ It has been suggested that the number of patients who will present with an acute allergy to CHG is likely to increase over time.^31,32^

The number of CHG-containing healthcare products in varied concentrations, including medical devices, hand soaps, body washes, surgical irrigation products, and oral rinses, also raises the specter of microbial selection, which may result in the emergence of resistant strains over time.^21–24,33^ Emerging CHG resistance has already been suggested in outbreaks of healthcare-associated infections in the United States, and high-frequency exposure to sublethal concentrations of CHG may enhance acquired resistance in organisms such as *Acinetobacter* spp, *K. pneumoniae*, and *Pseudomonas* spp, which are all known for their virulence and adaptability to antibiotics.^21^ Although the emerging of CHG resistance within the clinical setting is a current theoretical consideration, this perspective does not discount the benefits of continued research into the development of novel, effective, and safe skin antiseptic agents. The findings of this FDA phase 3 efficacy study support the need for continued diversification of our topical antiseptic armamentarium. ZuraGard combines a novel formulation of citrate ion in solution with alkyl parahydroxybenzoates, which adjunctively enhance the antimicrobial performance of the active ingredient isopropyl alcohol, maintaining stability and persistent bacterial log reductions on the surface of the skin.

In conclusion, the results of this large FDA phase 3 efficacy study demonstrate the effective antiseptic activity of ZuraGuard compared to Chloraprep, with no documented adverse effects. ZuraGard demonstrated an immediate and persistent antimicrobial efficacy, performing favorably compared to the current standard of care perioperative skin antiseptic agent, Chloraprep. ZuraGard effectively reduces the endogenous microbial populations associated with surgical wound contamination with the additional advantage of avoiding the risk of IgE-mediated anaphylaxis or potential microbial resistance. Further randomized, controlled clinical trials are warranted to assess the clinical efficacy of ZuraGard as an effective perioperative skin antiseptic agent for reducing the risk of surgical site infection across the spectrum of surgical disciplines.
